# Can the risk of obstetric anal sphincter injuries (OASIs) be predicted using a risk-scoring system?

**DOI:** 10.1186/1756-0500-7-471

**Published:** 2014-07-24

**Authors:** Karl C McPherson, Andrew D Beggs, Abdul H Sultan, Ranee Thakar

**Affiliations:** 1 Department of Obstetrics & Gynaecology, Croydon University Hospital, 530 London Road, Croydon, CR7 7YE London, UK; 2 Academic Department of Surgery, School of Cancer Sciences, Birmingham, UK

**Keywords:** Anal sphincter, Perineal trauma, Risk factors, Third and fourth degree tears

## Abstract

**Background:**

Perineal trauma involving the anal sphincter is an important complication of vaginal delivery. Prediction of anal sphincter injuries may improve the prevention of anal sphincter injuries. Our aim was to construct a risk scoring model to assist in both prediction and prevention of Obstetric Anal Sphincter Injuries (OASIs). We carried out an analysis of factors involved with OASIs, and tested the constructed model on new patient data.

**Methods:**

Data on all vaginal deliveries over a 5 year period (2004–2008) was obtained from the electronic maternity record system of one institution in the UK. All risk factors were analysed using logistic regression analysis. Odds ratios for independent variables were then used to construct a risk scoring algorithm. This algorithm was then tested on subsequent vaginal deliveries from the same institution to predict the incidence of OASIs.

**Results:**

Data on 16,920 births were analysed. OASIs occurred in 616 (3.6%) of all vaginal deliveries between 2004 and 2008. Significant (p < 0.05) variables that increased the risk of OASIs on multivariate analysis were: African-Caribbean descent, water immersion in labour, water birth, ventouse delivery, forceps delivery. The following variables remained independently significant in decreasing the risk of OASIs: South Asian descent, vaginal multiparity**,** current smoker, home delivery. The subsequent odds ratios were then used to construct a risk-scoring algorithm that was tested on a separate cohort of patients, showing a sensitivity of 52.7% and specificity of 71.1%.

**Conclusions:**

We have confirmed known risk factors previously associated with OASIs, namely parity, birth weight and use of instrumentation during delivery. We have also identified several previously unknown factors, namely smoking status, ethnicity and water immersion. This paper identifies a risk scoring system that fulfils the criteria of a reasonable predictor of the risk of OASIs. This supersedes current practice where no screening is implemented other than examination at the time of delivery by a single examiner. Further prospective studies are required to assess the clinical impact of this scoring system on the identification and prevention of third degree tears.

## Background

With the modernisation of intrapartum care, morbidity and mortality associated with vaginal delivery has reduced dramatically. However, Obstetric Anal Sphincter Injuries (OASIs) remain an important complication of vaginal delivery and its incidence appears to be rising [1]. While many women suffer no consequences, others develop varying degrees of flatus and faecal incontinence, which correlates to the degree of tear sustained [2]. Anal incontinence is an embarrassing condition that is under-reported by women and is associated with significantly reduced quality of life for affected women [3-5]. Sultan and Thakar reviewed 35 studies over a 25 year period and reported a mean prevalence of anal incontinence in 39% of women following a primary anal sphincter repair [6].

The incidence of OASIs in the literature varies widely between hospitals and countries [6-9], reflecting wide variations in obstetric practice and inaccurate reporting related to training of doctors and midwives [6,10]. Modifiable risk factors that have been shown previously to reduce OASIs include restricted use of episiotomy [10], medio-lateral instead of midline episiotomy [11], and preference of vacuum extraction to forceps delivery as the instrument of choice [12-16]. Under-diagnosis of anal sphincter injuries, particularly those involving the internal anal sphincter, remain a key cause for subsequent faecal incontinence [2]. There have been many associated features of labour that have been linked with the incidence of OASIs [12-16], however there remains little consensus on the causative risk factors related to OASIs.

Identification of these risk factors may inform future management of labour to reduce the overall incidence of OASIs. Anal sphincter injuries are known to cause significant morbidity and psychological distress [17]. Identification of risk factors may enable modification of labour and delivery practices, with a view to reduce the incidence of OASIs. A reduction in the incidence of OASIs would also reduce the likelihood of undiagnosed (missed) OASIs at the time of birth. These missed injuries are more likely to result in faecal incontinence, therefore making diagnosing all anal sphincter injuries at the time of vaginal delivery paramount [2,18].

The aims of this study were firstly to identify the incidence of OASIs in our population and to identify causal risk factors from data collected at the time of birth. We also aimed to use these risk factors to construct a risk-scoring system that can predict OASIs in a separate cohort of patients.

## Methods

### Study population

The study was carried out in a district general hospital based in the United Kingdom. Data was collected from the electronic maternity record system (Protos version 3.5) from 1st January, 2004 to 31st December 2008. Data was subsequently gathered according to the same criteria for deliveries from 2009–2011 for confirmation of the risk scoring model. Exclusion criteria were: caesarean delivery, birth prior to 24 weeks gestation, and multiple deliveries where one or more infants were delivered by caesarean section. Ethical approval was not necessary for this study as all patient identifiers were replaced with a unique patient code and the project did not meet the local criteria for ethical approval.

### Collection of data

In keeping with practice in the United Kingdom, all uncomplicated deliveries were managed by trained midwives. An obstetrician was called upon whenever required, for example if an instrumental or caesarean delivery or when an anal sphincter tear or other complications were suspected. The midwife in charge was responsible for entering and collecting information on pregnancy, labour and delivery on the electronic discharge system used to obtain this dataset.

### Variables

We collected information on the following maternal prenatal (age, vaginal parity, ethnicity) and obstetric factors (year of delivery, fetal presentation prior to labour, smoking status at booking, place of delivery, gestation at delivery, use of epidural or other analgesia water immersion during labour, duration of 2nd stage, mode of delivery, water birth, use of episiotomy shoulder dystocia, degree of perineal trauma, sex of infant, head circumference, birth weight,). Vaginal parity included only vaginal deliveries. Birth weight was recorded in grams, head circumference was measured in cm. OASIs was said to have occurred if there was any damage to the anal sphincters.

### Statistical analyses

The resultant data was analysed using Stata 11.2 (StataCorp, TX, USA). A logistic regression model was constructed using this information. Dichotomous variables were coded with 0 or 1 as baseline. For continuous normally distributed variables (age, birth weight), mean and standard deviation were calculated, and for continuous non-normally distributed variables (duration of 2nd stage), median and interquartile range were calculated. In order to model individual variable effects, separate univariate logistic regression models were carried out with obstetric anal sphincter injury (present = 1, absent = 0) as the independent variable and the factors used as the dependent variable. In order to correct for and model for the effects of interacting variables on each other, a multivariate logistic regression model was carried out using reverse stepwise model selection using an inclusion p-value of < 0.05. All variables used in the univariate analysis were carried through into the multivariate model. In order to study interactions between variables, likely interactions were modelled using a combination of t-tests, chi-squared testing and/or univariate logistic regression. The resulting odds ratios were used to calculate a risk-scoring algorithm that was used to test whether accurate prediction of OASIs could be achieved on a new cohort of deliveries. To do this, birth data was then collected on deliveries from the 1st of January 2009-31st of December 2011 within the same unit.

## Results

**Figure 1:**
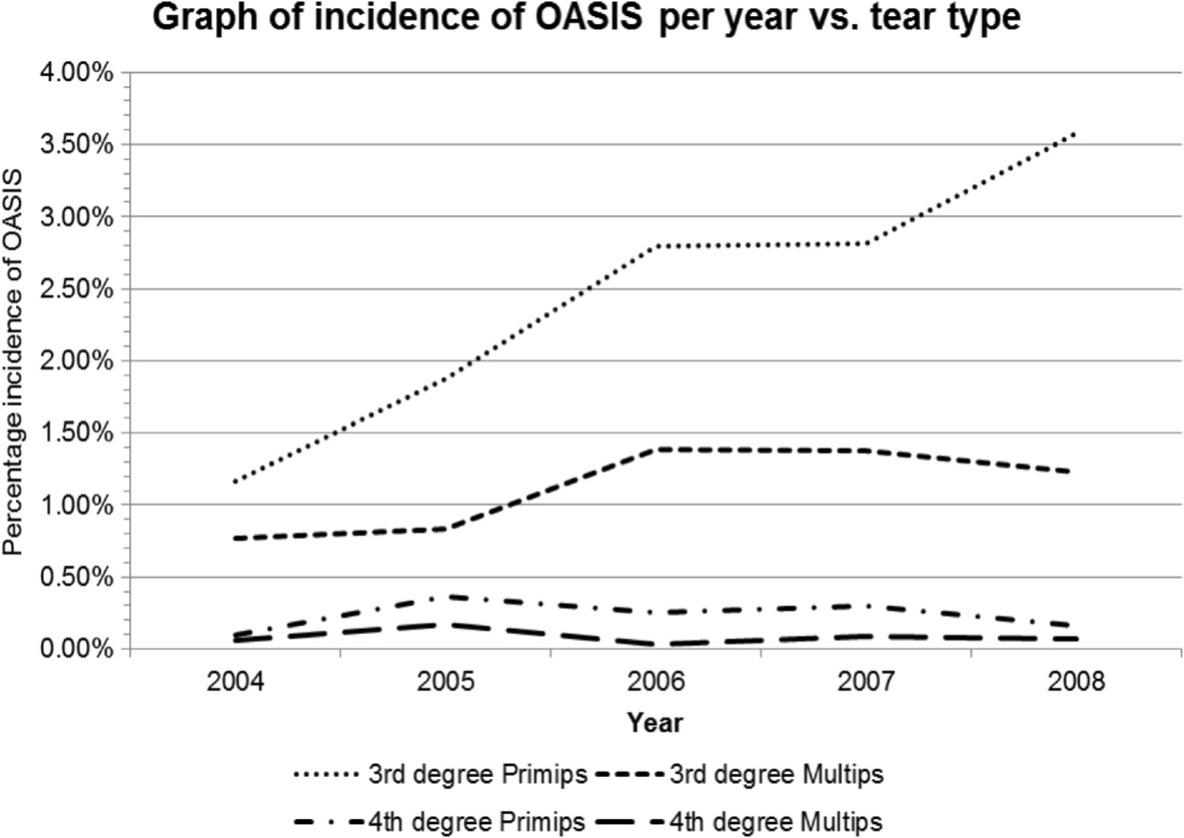
Graph of incidence of OASIS per year vs. tear type.

**Figure 2:**
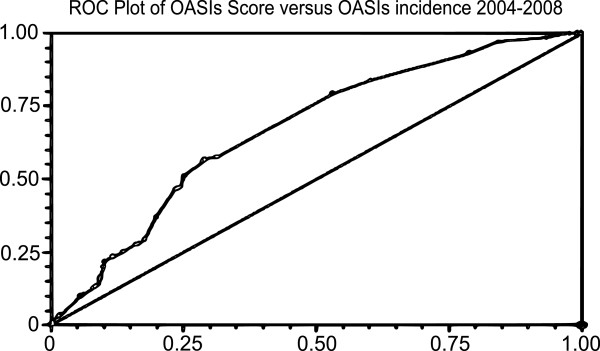
ROC curve demonstrating risk score relation to incidence of OASIS from 2004–2008.

**Figure 3:**
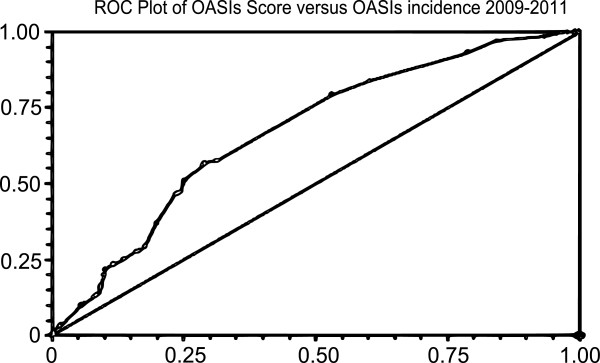
ROC curve demonstrating risk score relation to incidence of OASIS from 2009–2011.

Analysis was performed in 15,871 women who fulfilled the inclusion criteria with no missing data. OASIs occurred in 1040 of 26189 (3.97%) all vaginal deliveries. The of 3rd degree tear rate increased year on year particularly in primiparous women while the 4th degree tear rate remained static in both primips and multips (Figure 1). The demographic characteristics of these patients are shown in Table 1. Receiver-operator curves are shown for the two time points analysed (Figures 2 and 3).

**Table 1:** Table of data included in study

**Variable**	**N (%)**
Maternal age in years	28.9 years (SD +/- 6.0 )
Ethnicity	
Caucasian	7,369 (46.4%)
South Asian	1,681 (10.6%)
Afro-Caribbean	4,086 (25.8%)
East Asian	171 (1.1%)
Other	2,564 (16.2%)
Parity	
Nulliparous	6,568 (41.4%)
Para 1 greater than para 1	5,358 (33.8%) 3,945 (24.8%)
Smoking status	
Never smoked	12,499 (78.8%)
Ex-smoker	1,371 (8.6%)
Current smoker	2,001 (12.6%)
Place of delivery	
Hospital	15,350 (96.7%)
Home	454 (2.9%)
Other	67 (0.4%)
Mode of delivery	
Vaginal (non-instrumental)	14,313 (90.2%)
Forceps	372 (2.3%)
Ventouse	1,186 (7.5%)
Epidural	1,791 (11.3%)
Other analgesia	
None	15,647 (98.6%)
CSE	46 (0.3%)
Spinal	161 (1.0%)
Epidural	17 (0.1%)
Water immersion	714 (4.5%)
Duration of 2nd stage in minutes	Median 22 mins
	(IQR 10–54 mins)
Water birth	298 (1.9%)
Shoulder dystocia	262 (1.7%)
Mediolateral episiotomy	2,347 (14.8%)
Birth weight (grams)	3323 g (SD +/- 509)
Head circumference (cm)	33.9 cm (SD +/- 1.64)

The results of univariate analysis with OASIs as the independent variable are shown in Table 2. The following variables remained significant: forceps or ventouse delivery, use of mediolateral episiotomy, home birth, parity, ethnic category (“South Asian”, “Afro-Caribbean” & “East Asian”), birth weight, use of epidural, use of spinal anaesthetic, head circumference, presence of shoulder dystocia and current smoker at booking.

**Table 2:** Odds ratios from univariate regression model of OASIS vs. risk factors

**Variable**	**95% CI**	**OR**	**Subgroup**	**p-value**
Maternal age in years	0.99-1.02	1.01		0.415
Ethnicity	-	1	Caucasian	-
	1.75-2.74	2.19	South Asian	< 0.001
	0.60-0.95	0.76	Afro-Caribbean	0.019
	1.05-3.65	1.96	East Asian	0.035
	0.97-1.55	1.23	Other	0.082
Multiparity	0.34-0.45	0.39		< 0.001
Smoking status	-	1	Non-smoker	-
	0.77-1.35	1.01	Ex-smoker	0.891
	0.21-0.46	0.31	Smoker	< 0.001
Place of delivery	-	1	Hospital	-
	0.08-0.60	0.22	Home	0.003
	0.37-3.77	1.18	Other	0.779
Mode of delivery	-	1	Vaginal	-
	5.08-8.94	6.74	Forceps	< 0.001
	1.92-3.10	2.44	Ventouse	< 0.001
Epidural	-	1	No	-
	1.24-1.94	1.55	Yes	< 0.001
Other analgesia	-	1	None	-
	0.30-5.13	1.24	CSE	0.766
	5.59-11.87	8.14	Spinal	< 0.001
	0.23-12.89	1.71	Epidural	0.605
	1.42-2.62	1.93	Water immersion	< 0.001
Duration of 2nd stage in minutes	1.05-1.08	1.07		< 0.001
Water birth	0.74-2.20	1.27		0.378
Shoulder dystocia	-	1	No	-
	2.34-5.10	3.46	Yes	< 0.001
Mediolateral episiotomy	-	1	No	-
	2.14-3.08	2.57	Yes	< 0.001
Birth weight (grams)	1.0004-1.0008	1.0006		< 0.001
Head circumference (mm)	1.11-1.23	1.16		< 0.001

Multivariate reverse stepwise logistic regression analysis was then performed in order to allow for the effects of potentially interacting variables. The results are shown in Table 3. The following variables remained independently significant: African-Caribbean descent (OR 1.99, CI 1.69-2.34, p <0.001), ventouse delivery (OR 2.04, CI 1.69-2.46, p <0.001), forceps delivery (OR 5.62, CI 4.62-6.83, p <0.001), home delivery (OR 0.36, CI 0.19-0.68, p 0.001), current smoker (OR 0.35, CI 0.25-0.49, p <0.001), water birth (OR 1.46, CI 1.02-2.10, p0.041), water immersion in labour (OR 2.29, CI 1.78-2.94, p <0.001), vaginal multiparous (OR 0.77, CI (0.68-0.88, p <0.001), South Asian descent (OR 0.79, CI 0.66-0.93, p 0.007).

**Table 3:** Multivariate model of variables remaining independently significant as risk factors for OASIS

**p-value**	**95% CI**	**OR**	**Subgroup**	**Variable**
< 0. 001	1.61-3.75	2.45	Yes	Shoulder dystocia
				Mode of delivery
< 0. 001	2.25-4.35	3.13	Forceps	
0.001	1.18-1.99	1.54	Ventouse	
				Epidural
0.014	0.56-0.94	0.73	Yes	(Baseline = no)
				Place of delivery
0.039	0.13-0.95	0.35	Home	
				Other analgesia
< 0. 001	2.78-6.49	4.24	Spinal	
				Smoking status
0.008	0.38-0.87	0.58	Smoker	
0.002	1.22-2.32	1.68	Yes	Water immersion
< 0. 001	0.30-0.41	0.35		Multiparity
< 0. 001	1.03-1.06	1.04		Mothers age in years
				Ethnicity
< 0. 001	2.30-3.66	2.90	South Asian	
0.049	1.01-3.63	1.91	East Asian	
0.006	1.10-1.73	1.38	Other	
< 0. 001	1.000634-1.001007	1.000821		Birth weight in g

From this, the OASIs risk score was developed, using statistically significant variables from both antenatal and intrapartum events. Risk scores were set in accordance with the calculated odds ratios for each independently significant risk factor. Using this model, the optimal cut-off was predicted as 0.8, giving a sensitivity of 52.9%, specificity was 70.9%, area under curve was 0.636, 95% CI 0.609-0.655).

This model was then applied to delivery data from the same unit for deliveries from 2009–2011. 9458 deliveries met our exclusion criteria and were analysed. Using the same cut-off of 0.8, sensitivity was 52.7%, specificity was 71.1%, area under curve was 0.636 (95% CI 0.611-0.662), in keeping with the trend to prediction identified from the 2004–2008 data.

### Analysis of potentially interacting variables

In order to further study the effects of variables upon each other, a number of variables were re-analysed.

In order to ascertain whether the effect seen with water immersion was related to high frequency of instrumental delivery in this group, we compared these two variables. There were 714/15871 (4.5%) deliveries with water immersion of which significantly fewer, 29/714 (4.1%) underwent instrumental delivery versus 1529/15157 (10.1%) who did not undergo water immersion during labour but required instrumental delivery.

We analysed the relationship between parity and home birth status using univariate logistic regression. We found that patients undergoing home birth were significantly more likely to be multiparous (OR 1.87, 95% CI 1.68-2.09, p < 0.001). We then investigated the relationship between home birth and birth weight and found that in home births, mothers delivered significantly heavier babies (3450 g, 95% CI 3402-3498 g, p < 0.001, t-test) than those delivering in hospital (3320 g, 95% CI 3312-3328 g).

We then examined the association between maternal smoking status at booking with neonatal birth weight. There was a significant difference between the two groups, with non-smokers delivering infants of an average weight of 3344 g, whereas smokers delivered infants weighing 3178 g (p <0.0001).

## Discussion

In this large retrospective study we demonstrated that forceps delivery, ventouse delivery, African-Caribbean descent, water immersion and water birth independently increased the incidence of OASIs. Home birth, smoking, vaginal parity greater than zero and South Asian descent were protective against OASIs. Several studies have cited instrumental delivery as a significant risk factor for OASIs [14,19,20]. The data in this study continues to support this, with delivery particularly by forceps, and to a lesser extent, ventouse delivery posing the greatest risk to the perineum [14]. The cause for this discrepancy between risk of OASIs between ventouse and forceps has yet to be understood. One opinion is that this observed variation is due to the indication for use of forceps in preference to ventouse in more difficult assisted vaginal deliveries and thereby increasing the force applied to the perineum. This may also explain the association with forceps delivery with both pelvic organ prolapse and stress incontinence, in keeping with the idea of greater soft tissue injury with the use of forceps [19-21]. Other suggestions include increased risk as a result of decreased operator skill with forceps delivery or the design of the shaft and blades of the forceps in comparison to the handle of the ventouse and how the perineum is stretched with the delivery of the fetal head.

Due to the mixed ethnicity of our population we were able to study the effect of this on OASIs. Our study indicates that African-Caribbean women are at an increased risk of anal sphincter injuries. South Asian women had independently decreased risks of OASIs in comparison to their Caucasian and Afro-Caribbean counterparts. This contradicts the findings by Dua et al. where data in relation to perineal body length and ethnicity was first described in a single unit [22]. In their study, ethnicity was reported according to the criteria of the National Statistics Classification of either Asian or Asian British, but there is no mention as to whether this information was self-reported. Only 16 patients were from either African-Caribbean or Chinese descent and normative data was not reported for these ethnicities. It is possible that this observed variation is mediated by a different “Asian” population mix, given in our study this term was used to describe patients from India, Pakistan, Bangladesh and Sri Lanka. Further studies investigating the respective incidences of OASIs from descendants from each of these countries are required to prove or disprove these findings.

Regarding the significance of water immersion and water birth and its relationship to OASIs, Samuelsson et al. [23] has previously suggested prospectively that perineal oedema is significantly associated with perineal lacerations. It is also clear that the positioning of the mother during water immersion precludes surveillance of the perineum that in advanced labour may lead to unrecognised tears during birth. Samuelsson also confirmed poor visualisation of the perineum at the time of birth as a causative factor in OASIs. To confirm this, prospective analysis of the duration of water immersion and cervical dilatation on leaving the pool prior to vaginal delivery affects the rate of OASIs is needed. Our study suggests that water immersion may be associated with increased rates of OASIs, therefore potentially negating the pain relieving benefits of water immersion [24].

Maternal multiparity has been previously demonstrated as a decreased risk for OASIS [10,13-16,24]. Ultrasonographical studies suggest that following vaginal delivery, the genital hiatus dimensions are increased, and this change reduced the risk of OASIs to a decrease in the rigidity of the pelvic floor and perineal tissues [21,25].

We also found that smoking at booking in pregnancy was strongly negatively correlated to OASIs [26]. The most plausible reason for this is that smokers tend to give birth to smaller babies because of a direct effect of smoking on fetal growth as demonstrated in this paper of a 166 g reduction in birth weight [26,27]. This study however contrasts this somewhat, as birth weight was not an independent factor in OASIs. Given the far-reaching implications of smoking on pregnancy including the increased risk of stillbirth, smoking in pregnancy should still be actively discouraged [27].

Place of delivery was the most protective of all risk factors examined by this study. Given the specific selection criteria for home births and intrapartum risk identification in order to transfer women with new complications to hospital to complete their delivery, any interpretation of this data must be counterbalanced by this bias. We found that mothers undergoing home birth were significantly likely to be of higher parity (which would reduce OASIs risk) but had significantly heavier babies in keeping with other publications [28,29]. In this study there were low numbers of home births and further research is needed to see whether this effect can be generalised to all patients, but it is likely that the effect of home birth is likely to be due to selection bias rather than any biological effect.

We are not aware of another successful OASIs risk scoring system in the literature. Our scoring system demonstrates a specificity of approximately 71% reliably across both datasets, showing a low-risk score was associated with a low risk of OASIs. However the sensitivity remained low across both cohorts, indicating a low chance of a high-risk result indicating anal sphincter injury. At present the OASIs score is a reasonable predictor of anal sphincter injury, but may be improved by parameters not recorded in this dataset such as perineal body length [22,23,29].

In our study, several previously identified risk factors e.g. birth weight, maternal age, shoulder dystocia, use of right mediolateral episiotomy duration of second stage and mediolateral episiotomy [12,13,16] were not identified as independent risk factors for OASIs by this study.

### Bias and validity

As a retrospective study, there are likely to be influences on the results observed beyond those recorded in the discharge data [30]. Our study is however strengthened by the heterogeneous, multi-ethnic population examined, that differs from previous publications in relation to the incidence of OASIs.

There are several potential weaknesses to our study. The accuracy of the onset of the second stage of labour depends on when vaginal examination to confirm full dilatation of the cervix. Ethnicity was interpreted according to self-declaration and therefore was open to reporter bias. The indication for instrumental delivery was not recorded and may yield further information as to the cause for the discrepancy in the incidence of OASIs between forceps and ventouse. Double instrumentation was not recorded in this dataset.

## Conclusions

There are several factors that control the risk of OASIs. The main themes that affect the incidence of OASIs are maternal perineal soft tissue condition prior to delivery and force applied to the perineum. Instrument choice at assisted deliveries and restricted access to birthing pools in labour appears to be the most readily modifiable risks for OASIs.

The incidence of OASIs may be modifiable according to these findings, particularly with regards to the choice of instrument at instrumental delivery and use of water immersion and water birth in labour. This study confirms that intrapartum care can both increase and decrease the risk of anal tears. This study suggests OASIs risk scoring may be a means of screening for those at risk of anal sphincter injury, and future research is required to identify if for instance, a second examination for those who have a positive score and perineal injury improves detection of anal sphincter injuries as previously confirmed by Andrews et al. [18]. As a preliminary tool, use of the OASIs score also offers potential to assess interventions to avoid anal sphincter trauma such as perineal support for those with a high-risk result prior to delivery [31]. The OASIs score gives us an algorithm for understanding how independent factors related to anal sphincter trauma interact and may highlight strategies for reducing the incidence of OASIs. For any risk-scoring system to be clinically-applicable at a global level in relation to anal sphincter injuries, further prospective, multi-centre trials are required. Further prospective data not recorded in this cohort may improve the predictive ability of the OASIs risk score [2,10].

## Competing interests

The authors declare that they have no competing interests.

## Authors’ contributions

AB and KMcP carried out the statistical analysis; AB, KMcP, RT and AS collected the data; AB, KMcP, RT and AS wrote the manuscript. All authors read and approved the final manuscript.
